# Characterization of *Francisella tularensis* Schu S4 mutants identified from a transposon library screened for O-antigen and capsule deficiencies

**DOI:** 10.3389/fmicb.2015.00338

**Published:** 2015-05-05

**Authors:** Jed A. Rasmussen, Joshua R. Fletcher, Matthew E. Long, Lee-Ann H. Allen, Bradley D. Jones

**Affiliations:** ^1^Department of Microbiology, University of Iowa Carver College of MedicineIowa City, IA, USA; ^2^Genetics Program, University of Iowa Carver College of MedicineIowa City, IA, USA; ^3^Molecular and Cellular Biology Program, University of Iowa Carver College of MedicineIowa City, IA, USA; ^4^Department of Internal Medicine, University of Iowa Carver College of MedicineIowa City, IA, USA

**Keywords:** *Francisella tularensis*, O-antigen, capsule, transposon mutagenesis, innate immunity, intracellular growth, mouse virulence

## Abstract

The lipopolysaccharide (LPS) and O-antigen polysaccharide capsule structures of *Francisella tularensis* play significant roles in helping these highly virulent bacteria avoid detection within a host. We previously created pools of *F. tularensis* mutants that we screened to identify strains that were not reactive to a monoclonal antibody to the O-antigen capsule. To follow up previously published work, we characterize further seven of the *F. tularensis* Schu S4 mutant strains identified by our screen. These *F. tularensis* strains carry the following transposon mutations: *FTT0846*::Tn5, *hemH*::Tn5, *wbtA*::Tn5, *wzy*::Tn5, *FTT0673p*/*prsA*::Tn*5*, *manB*::Tn5, or *dnaJ*::Tn5. Each of these strains displayed sensitivity to human serum, to varying degrees, when compared to wild-type *F. tularensis* Schu S4. By Western blot, only *FTT0846*::Tn5, *wbtA*::Tn5, *wzy*::Tn5, and *manB*::Tn5 strains did not react to the capsule and LPS O-antigen antibody 11B7, although the *wzy*::Tn5 strain did have a single O-antigen reactive band that was detected by the FB11 monoclonal antibody. Of these strains, *manB*::Tn5 and *FTT0846* appear to have LPS core truncations, whereas *wbtA*::Tn5 and *wzy*::Tn5 had LPS core structures that are similar to the parent *F. tularensis* Schu S4. These strains were also shown to have poor growth within human monocyte derived macrophages (MDMs) and bone marrow derived macrophages (BMDMs). We examined the virulence of these strains in mice, following intranasal challenge, and found that each was attenuated compared to wild type Schu S4. Our results provide additional strong evidence that LPS and/or capsule are *F. tularensis* virulence factors that most likely function by providing a stealth shield that prevents the host immune system from detecting this potent pathogen.

## Introduction

*Francisella tularensis* is the causative agent of the human disease tularemia. This small Gram negative organism is able to cause disease with as few as 10 colony forming units (CFU) when inhaled, and without treatment, upwards of 60% of those infected may die (Saslaw et al., 1961; Tarnvik and Berglund, 2003; Oyston, 2008). Due to the ease of aerosolization, low infectious dose, and potentially high fatality rates, the Center for Disease Control and Prevention (CDC) has classified *F. tularensis* has a Tier I select agent (Center for Disease Control and Prevention, 2013). Although, there are relatively few naturally acquired *Francisella* infections per year in the United States (Carvalho et al., 2014), a primary concern is that the organism will be used in a bioterrorist attack. This is not an unfounded concern as *Francisella* species were weaponized by Japan, United States, and the former U.S.S.R., during and after World War II (Oyston et al., 2004; Sjostedt, 2007). These concerns have highlighted the need to develop a vaccine to prevent disease and associated deaths from the potential use of *Francisella* as a bioterrorist weapon.

Although essential genes for bacterial growth and replication have been identified as important for *Francisella* to cause productive infection, few other genes have been clearly linked to pathogenesis. Our studies of *F. tularensis waaY* and *waaL* mutants have previously demonstrated that LPS associated O-antigen and capsule have significant roles in the pathogenesis of *F. tularensis* Schu S4 (Lindemann et al., 2011; Rasmussen et al., 2014). These genes function in LPS assembly by participating in the LPS core construction (*waaY*) and in ligating the tetrasaccharide O-antigen repeats to LPS core (*waaL*) (Rasmussen et al., 2014). In addition, a number of other labs have shown that strain with mutations in genes that are involved in capsule and LPS biosynthesis have significantly altered virulence properties (Sandstrom et al., 1988; Comstock and Kasper, 2006; McLendon et al., 2006; Barker and Klose, 2007; Su et al., 2007; Weiss et al., 2007; Apicella et al., 2010). Strains carrying mutations in either of these genes enter into human MDMs at elevated levels, are cytotoxic for MDMs at early time points, and are much more sensitive to human serum than the parent Schu S4 strain (Lindemann et al., 2011). These data suggest that one of the significant functions of the O-antigen structures of both the *Francisella* LPS and capsule are to provide stealth concealment for the organism, allowing it to avoid detection by the host. This is not a novel strategy to avoid host recognition, as it has been described in some detail for other Gram-negative organisms (Comstock and Kasper, 2006). Interestingly, even though the *waaY* or *waaL* mutants lack O-antigen and capsule and are hypercytotoxic in MDMs (Lindemann et al., 2011), these strains still disseminate to the livers and spleens of mice during infection but cause death by a different mechanism than wild type *F. tularensis* (Rasmussen et al., 2014). These data imply that *F. tularensis* expresses antigens that can induce host inflammation, but that they are obscured by the LPS and capsule in wild type organisms. The virulence still possessed by the *F. tularensis* O-antigen mutants is unusual since O-antigen mutants in other species of bacteria are typically avirulent (Rietschel, 1984; Iredell et al., 1998; Prior et al., 2003; Ho and Waldor, 2007; Sheng et al., 2008; Post et al., 2012).

An interesting aspect of the *F. tularensis* capsule is that the polysaccharide repeat structure is exactly the same as the LPS O-antigen (Vinogradov et al., 2002; Thomas et al., 2007; Apicella et al., 2010; Wang et al., 2011). While the O-antigen structure is the same, *F. tularensis* capsule is assembled without core LPS sugars, 2-keto-3-deoxyoctulsonic acid (KDO), or lipid A (Apicella et al., 2010). Due to the identical tetrasaccharide repeat containing acetimido sugars and high molecular weight structure, *F. tularensis* capsule is considered to be a Group 4 capsule (Whitfield, 2006). Studies of Group 4 capsules in other organisms have revealed distinct LPS and capsule biosynthetic pathways (Whitfield, 2006). To date, however, divergent biosynthetic pathways for *F. tularensis* LPS and capsule have not been discovered. Although extensive analysis of O-antigen biosynthesis has not been done in *Francisella*, LVS strains with mutations in the *wbt* genes do not produce O-antigen (Raynaud et al., 2007). In addition, *wbt* mutant strains are also more susceptible to complement-mediated lysis than *F. tularensis* LVS (Li et al., 2007; Clay et al., 2008). Strains carrying either a *waaY* or *waaL* mutation display moderate attenuation in an intranasal infection (i.n.) as seen by the 1000-fold and 100-fold increase in the LD_50_, respectively (Rasmussen et al., 2014), compared to the *F. tularensis* Schu S4 theoretical LD_50_ of ~1 CFU (Kim et al., 2012). When infected intraperitoneally (i.p.) the mutants are significantly attenuated (more than a 100,000 fold increase in comparison to the wild type LD_100_ of ~1 CFU), as would be expected for traditional *waa* mutants (Rietschel, 1984).

We screened a *F. tularensis* Schu S4 mutant library of ~7500 mutants by enzyme-linked immunosorbent assay (ELISA) to identify strains defective for capsule production (Rasmussen et al., 2014). From that screen we identified six strains with mutations in genes *FTT0846*, *FTT1138* (*hemH*), *FTT1236* (*waaY*), *FTT1238c* (*waaL*), *FTT1464c* (*wbtA*), and *FTT1458c* (*wzy*) that had significantly less capsule compared to the parent strain, as detected by a lack of binding to the anti-capsule mAb (11B7) (Rasmussen et al., 2014). Mutation of these genes also caused decreased binding of the anti-O-antigen mAb (FB11) as detected by ELISA (Rasmussen et al., 2014). In a second screen, we identified three additional mutant strains *FTT1447c* (*manB*), *FTT1512c* (*dnaJ*) and an intergenic insertion between *FTT0673* and *FTT0674c* (*prsA*) that also had decreased amounts of detectable capsule.

Although we have previously characterized the *F. tularensis waaY* and *waaL* mutants in some detail, this manuscript describes our work characterizing the additional *F. tularensis* strains with O-antigen defects that map to *dnaJ*, *hemH*, *manB*, *wbtA*, *wzy*, *FTT0846*, and *FTT0673p*/*prsAp*::Tn5. We present data on the serum sensitivity of these strains, characteristics of the capsule/O-antigen and LPS core of each strains, the ability to grow in murine bone marrow derived macrophages and human macrophages, and attenuation for virulence in a murine infection model. We have found that mutations in *dnaJ*, *hemH*, and the *FTT0673p*/*prsAp*::Tn5 strain did not have significant differences in many of the assays performed, whereas the strains with mutations in *manB*, *wzy*, *wbtA*, and *FTT0846* display phenotypes similar to those observed for the *F. tularensis waaY* and *waaL* mutants.

## Materials and methods

### Bacterial strains, plasmid construction, and growth conditions

*F. tularensis* subsp. *tularensis* Schu S4 was routinely cultured on Modified Mueller Hinton (MMH) plates (Acumedia, Lansing, MI) or in MMH broth. Schu S4 mutant strains were grown on MMH plates supplemented with 50 μg/ml of kanamycin as needed. Complementation was performed by cloning the coding sequence of a gene downstream of the constitutive P*_gro_* promoter into pTrc99A. Primers used to amplify functional *wzy* and *manB* genes for complementation are respectively, *wzy*: Forward, 5′-GGATCCGGTACCGTGTACATAAAAAAAGTGTCTTTTAAAATT-3′ and Reverse, 5′-GTCGACAAGGTTTATTATTAAATGTACAAACC-3′. *manB*: Forward, 5′-GGATCCGGTACCATGAGACAAACTATAATAAAAGAAATAATC-3′ and Reverse, 5′-GTCGACAGAAAGTTAGGGAATATTTTTGACTG-3′. The promoter-gene construct was then cloned into the *Francisella* shuttle vector, pBB103 (Buchan et al., 2008) and cryotransformed into mutant strains as previously described (Buchan et al., 2008). Complemented strains were grown on MMH plates or in MMH broth supplemented with 50 μg/ml of spectinomycin. All work with *F. tularensis* Schu S4 was performed within the Carver College of Medicine Biosafety Level 3 (BSL3) Core Facility and experimental protocols were reviewed for safety by the BSL3 Oversight Committee of The University of Iowa Carver College of Medicine. Recombinant DNA work with *F. tularensis* Schu S4 was reviewed and approved by the University of Iowa Institutional Biosafety Committee.

### *In vitro* growth assay

To determine whether *F. tularensis* mutant strains exhibited growth defects compared to wild type Schu S4, wild type and mutants were grown to saturation with shaking at 37°C and diluted into fresh MMH broth to an OD_600_ of 0.1. Broth cultures were shaken at 200 rpm at 37°C, and the optical density was determined at intervals. The doubling time (*T*) was calculated using the formula *N* = *N^kt^*_0*e*_, where *T* = (ln 2)/*k*, and growth indices were calculated by taking the same time points in the linear range for comparison (*t* is time, *N* is the amount after *t*, *N_0_* is the initial amount, and *k* is the constant rate of growth).

### Serum sensitivity assay

After obtaining informed consent, human serum was obtained from 15 to 20 individuals with no known history of tularemia and then pooled following the protocol (#200307026) approved by the Institutional Review Board (IRB) for human subjects of the University of Iowa. Bacteria were grown in MMH broth (with 50 μg/ml of spectinomycin for complemented mutants) for ~18 h at 200 rpm at 37°C. Bacteria were quantitated by measuring the OD_600_. Bacteria were added to PBS to make a culture of 1 × 10^7^ CFU/ml in 50% pooled normal human serum and incubated with shaking at 37°C for 90 min. Before and after incubation, bacteria were serially diluted in PBS, plated on MMH plates (with spectinomycin as needed), and grown for 2 days at 37°C with 5% CO_2_. Strains were serially diluted and plated for inputs and also after serum treatment to determine percentage of survivability.

### Page, immunoblotting and Emerald green stain of bacterial whole cell lysates

*Francisella* strains from freshly streaked agar plates were inoculated into MMH broth and grown for 24 h before the OD_600_ was measured and recorded. One ml broth cultures were centrifuged at 8000x g for 1 min before the pellet was resuspended in Buffer Part A, [6 mM Tris, 10 mM EDTA, and 2% (wt/vol) SDS (pH 6.8)] and heated to 65°C to sterilize cultures. Bacterial lysates were incubated with proteinase K (New England Biolabs, Ipswitch, MA) at 37°C for 24 h before lyophilizing. Approximately 5 μg of bacterial material from each sample was mixed with NuPage (Life Technologies, Carlsbad, CA) sample reducing agent and buffer, boiled for 10 min, and loaded into a 4–12% Bis-Tris NuPage gel and separated by electrophoresis using NuPage MES SDS running buffer (Life Technologies, Carlsbad, CA). For immunoblots, samples separated by PAGE were transferred to nitrocellulose using a semi-dry transfer system (Bio-Rad, Hercules, CA). The nitrocellulose membrane was blocked in blocking buffer (5% BSA in PBS) and then incubated with either primary mAb 11B7 (Dr. Apicella, University of Iowa, IA) to detect capsule, or mAb FB11 to detect LPS O-antigen (QED Bioscience, San Diego, CA). Bands were visualized using goat anti-mouse IgG (H + L) conjugated to horseradish peroxidase (Jackson ImmunoResearch, West Grove, PA) and SuperSignal West Pico chemiluminescent substrate (Pierce Biotechnology, Rockford, IL). The ProQ-Emerald 300 stain was used to visualize carbohydrates and the protocol was followed according to the instructions of the manufacturer (Invitrogen, Waltham, MA).

### Isolation and culture of human and murine macrophages

Venous blood was drawn from healthy adult volunteers using a protocol approved by The University of Iowa Institutional Review Board (IRB) for Human Subjects at the University of Iowa, and all subjects provided informed consent. Peripheral blood mononuclear cells (PBMCs) were isolated as described previously (Schulert and Allen, 2006). Briefly, PBMCs were isolated from venous blood by centrifugation on Ficoll-Hypaque (GE Healthcare, Piscataway, NJ), washed twice in HEPES-buffered RPMI 1640 with L-glutamine (RPMI) (Lonza, Walkersville, MD), seeded into Teflon jars at 2 × 10^6^ cells/ml, and allowed to differentiate into MDMs for 5 to 7 days in RPMI 1640 plus 20% autologous serum at 37°C with 5% CO_2_.

To isolate BMDMs, 6–8 week old BALB/c female mice were purchased from Frederick National Cancer Institute (NCI, Frederick, MD) and femurs were harvested and flushed with Dulbecco's modified Eagle medium (DMEM, Life Technologies, Carlsbad, CA). Cells were grown in DMEM with 20% L929 cell-conditioned media, 10% heat-inactivated fetal bovine serum (FBS, Life Technologies, Carlsbad, CA) and supplemented with 100 μg/ml of streptomycin and 100 U/ml of penicillin. Cells were incubated at 37°C with 5% CO_2_ for 5–7 days until monolayers of macrophages were detected.

### Human and murine intramacrophage growth

To quantify the extent of intracellular replication, MDMs were plated in Costar 24-well dishes (Sigma-Aldrich, St. Louis, MO) at a density of 1–2×10^5^ cells/well and allowed to adhere overnight at 37°C. Monolayers were washed with warm PBS twice, and infected in duplicate or triplicate with unopsonized wild-type Schu S4 or mutant strains in RPMI plus 2.5% heat-inactivated (56°C, 30 min) pooled human serum at an MOI of 100:1. After 1 h at 37°C, monolayers were washed three times with PBS to remove extracellular bacteria, and fresh RPMI plus 2.5% heat inactivated normal human serum was added. Wells were lysed in 0.5% saponin at 1, 16, and 24 h post-infection. Lysates were serially diluted in PBS, and viable bacteria were enumerated by plating on MMH agar as described above. BMDM infections using the appropriate media were done similarly to the human MDM infections. BMDMs were plated at a density of 3 × 10^5^ cells/well and infected at an MOI of 100:1.

### Confocal microscopy

Macrophages infected with *F. tularensis* were processed for microscopic analysis as previously described (Allen and Aderem, 1996) with minor modifications. Cells were fixed in 10% formalin, permeabilized by the addition of -20°C acetone and methanol (1:1), and blocked at 4°C for 5 days in PBS plus 0.5 mg/ml sodium azide, 5 mg/ml BSA (Thermo Fisher Scientific, Waltham, MA) and 10% horse serum (Thermo Fisher Scientific, Waltham, MA). Parallel samples were lysed and examined for sterility so that experimental samples could be removed from the BSL-3 facility. The rabbit anti-*F. tularensis* antiserum (BD Diagnostics, Franklin Lakes, NJ) and Dyelight 549-conjugated goat anti-rabbit IgG F(ab')_2_ secondary antibody from Jackson Immuno Research Laboratories (West Grove, PA) were used to detect wild type and mutant strains. The late endosome/lysosome-associated membrane protein-1 (lamp-1) was detected using a mouse anti-human lamp-1 monoclonal antibody (H4A3) from the Developmental Studies Hybridoma Bank at the University of Iowa (IA, Iowa City) and Dyelight 488-conjugated goat anti-mouse IgG F(ab')_2_ secondary antibody (Jackson Immuno Research, West Grove, PA). Images were obtained using a Zeiss LSM-510 confocal microscope using Zen software (Carl Zeiss Inc., Thornwood, NY).

### Murine infections and organ burden

BALB/c female mice, 6–8 weeks of age, were purchased from NCI for all sets of animal experiments. Groups of five mice were used for evaluation of virulence (determining the LD_50_) of *Francisella* strains and groups of six mice were used for experiments in which bacterial growth in murine organs was examined. Mice anesthetized with isoflurane (Sigma-Aldrich, St. Louis, MO) vapor were infected intranasally with 50 μl inocula mixed in PBS and were monitored for up to 26 days post-infection for the virulence studies. Doses ranging from 10^1^ to 10^5^ CFU were estimated from OD_600_ readings and were confirmed by serial dilution and plating of the bacterial suspension. For determination of bacterial burden in organs, mice were infected as described above, with mutant strains at approximately 5- 20-fold lower than the LD_50_ dose for each strain. Lungs, livers and spleens were harvested 40 days post-infection. Organs were homogenized using 50 mL tissue grinders (Thermo Fisher Scientific, Waltham, MA) in 2 ml of 1% saponin (ACROS, New Jersey). Organ homogenates were serially diluted in PBS and plated to enumerate the bacterial load per organ. All work with *F. tularensis* Schu S4 was performed within the Carver College of Medicine Biosafety Level 3 (BSL3) Core Facility, and all experimental protocols were reviewed for safety by the BSL3 Oversight Committee of the University of Iowa Carver College of Medicine. Recombinant DNA work with *F. tularensis* Schu S4 was approved by the Institutional Biosafety Committee.

## Results

### Identification of *F. tularensis* genes involved in capsule and O-antigen synthesis

**Figure 1:**
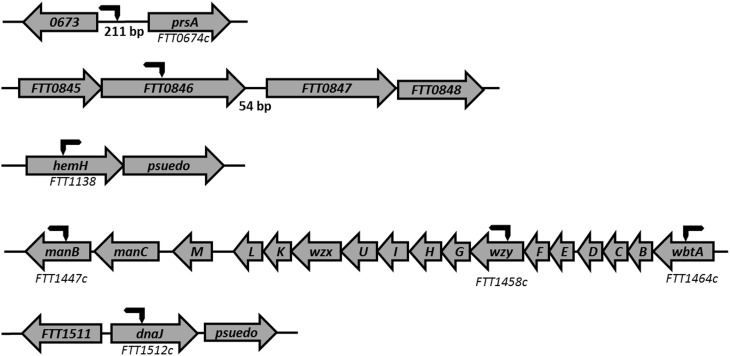
**Chromosomal location of Tn5 insertions**. Seven mutant strains that were disrupted in genes important for capsule production, were identified from the screened Schu S4 library using the capsule mAbs 11B7 and 10E8. Rescue cloning was performed and the plasmids obtained were sent to The University of Iowa School of Medicine DNA Core for sequencing. A Tn*5* primer was used to sequence the *F. tularensis* chromosomal DNA flanking the Tn*5* transposon insertion. One end of the arrow shows the approximate site of transposon insertion into the chromosome and the arrow to the left or right shows the direction of the transposon element.

A total of nine strains were identified by screening a Tn*5* transposon library of *F. tularensis* Schu S4 with monoclonal antibodies produced against *F. tularensis* capsule preparations. The mutations in three strains, *FTT0673p*/*prsA*::Tn*5*, *manB*, and *dnaJ*, were identified as important in capsule biosynthesis as the strains displayed reduced binding to the mAb 10E8. In a second screen, using high affinity mAb 11B7, six strains with mutations in *FTT0846*, *hemH, waaY*, *waaL*, *wbtA*, or *wzy* were independently identified as having a role in capsule biosynthesis. The chromosomal locations of the genes were identified by rescue cloning of the transposon insertion site and sequencing of the flanking chromosomal DNA (Rasmussen et al., 2014). The sites of the transposon insertions are shown in Figure 1. The *FTT0673*, *prsA*, *FTT0846*, and *hemH* genes that we have identified here have not been targeted for mutation or identified in previous genetic screens of *Francisella*. However, the genes *waaY* (Weiss et al., 2007; Lindemann et al., 2011; Case et al., 2013), *waaL* (Maier et al., 2007; Weiss et al., 2007; Lindemann et al., 2011), *wbtA* (Maier et al., 2007; Su et al., 2007), *wzy* (Lindemann et al., 2011), and *manB* (Weiss et al., 2007; Lindemann et al., 2011; Case et al., 2013) genes have been previously identified as important for *Francisella* virulence. Although the *dnaJ* gene has not been identified previously as important in virulence, its transcription has been reported to increase during *F. tularensis* intracellular growth (Wehrly et al., 2009). Growth rates were determined for all of the mutant strains and were found to be similar to wild type Schu S4 (Figure S1).

### Characterization of the LPS and capsule structures of *F. tularensis* mutants

**Figure 2:**
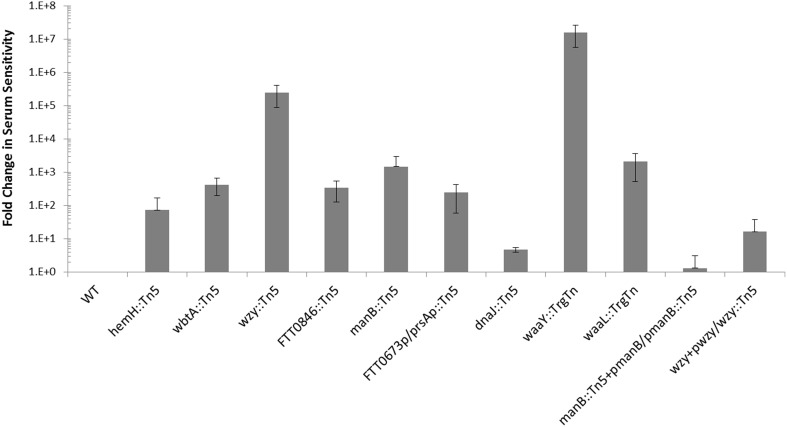
**Comparison of serum sensitivity of mutants to wild type Schu S4**. The serum sensitivity of *F. tularensis* mutants and complemented mutants is compared to wild type and is displayed as fold-change. Data were normalized to wild type. *F. tularensis* strains were prepared identically, exposed to pooled human serum for 90 min and then serially diluted.

We have shown previously that mutations in some *F. tularensis* genes important for O-antigen and capsule biosynthesis result in sensitivity to human serum complement (Lindemann et al., 2011). Serum sensitivity was tested for the *F. tularensis* strains with mutations in *dnaJ*::Tn*5*, *hemH*::Tn*5*, *manB*::Tn*5*, *wbtA*::Tn*5*, *FTT0846*::Tn*5*, or *FTT0673p*/*prsAp*::Tn*5* by exposing them to 50% pooled human serum for 90 min at 37°C. Each of the mutants displays some degree of serum sensitivity when compared to wild type (Figure 2). The fold increase in serum sensitivity of each *F. tularensis* mutant strain, compared to wild type was ~5 for *dnaJ1*::Tn5, ~70 for *hemH*::Tn5, ~245 for *FTT0673p*/*prsAp*::Tn5, ~330 for *FTT0846*, ~425 for *wbtA*::Tn5, ~1.5 × 10^3^ for *manB*::Tn5, ~2 × 10^3^ for *waaL*::TrgTn, 2.4 × 10^4^ for *wzy*::Tn5, and ~1.6 × 10^7^ for *waaY*::TrgTn. These results provided further evidence that the mutated genes are involved in LPS biosynthesis, capsule biosynthesis, or biosynthesis of an outer membrane component since these structures are known to affect serum sensitivity. An effort was made to complement each of the mutant strains back to a wild type phenotype but we were only able to restore functions in the *wzy* and *manB* mutants. Complementation of the *wzy* or the *manB* mutation restored serum resistance ~ 500- and ~8.5 × 10^3^-fold, respectively (Figure 2). Difficulty in complementing mutants in *Francisella* has been reported by several other *F. tularensis* research groups (Maier et al., 2004; Zogaj and Klose, 2010; de Bruin et al., 2011).

**Figure 3:**
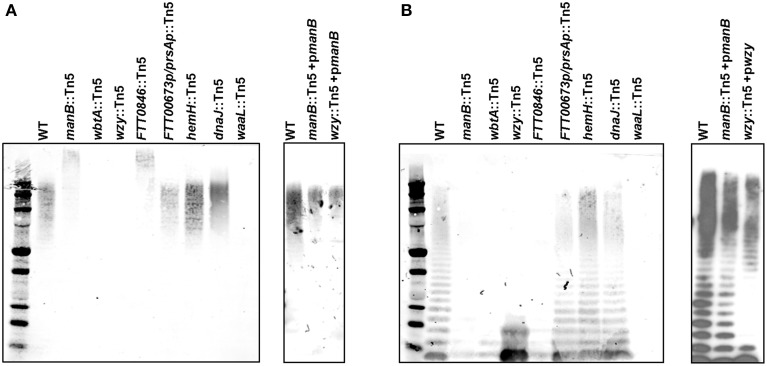
**Capsule and LPS O-antigen phenotypes of mutant strains**. Bacteria were grown in MMH broth for ~24 h and lysed with Buffer Part A at 65°C. **(A)** The anti-capsule mAb (11B7) was used to detect capsule in whole cell lysates of wild type *F. tularensis*, O-antigen mutants, and complemented mutant strains by immunoblotting. **(B)** To detect LPS in immunoblots of whole cell lysates of wild type, mutants, and complemented mutants using the anti-O-antigen mAb (FB11).

To more thoroughly characterize the LPS and capsule produced by each mutant, whole cell lysates were prepared from each strain and treated with proteinase K. The lysates were run on an SDS-PAGE gel, transferred to nitrocellulose, and probed with the anti-O-antigen (FB11) and anti-capsule antibodies (11B7). The anti-capsule immunoblot revealed that both the *wbtA* and *wzy* mutants lacked detectable capsule whereas the *FTT0846* and *manB* mutants had altered capsule species that were of higher molecular weight than wild-type Schu S4 capsule. Additionally, the altered capsules were less abundant than that of wild type Schu S4. The *dnaJ*::Tn*5*, *hemH*::Tn*5*, or *FTT0673p*/*prsAp*::Tn*5* mutants appeared to produce capsule in similar amounts to the parent strain (Figure 3). The anti-O-antigen immunoblot revealed that the *wbtA*, *FTT0846*, and *manB* mutants did not show LPS O-antigen laddering, whereas the *wzy* mutant produced a truncated LPS associated O-antigen structure, containing just one or two O-antigen subunits. This latter phenotype has been previously reported for an LVS *wzy* mutant (Kim et al., 2010). The *dnaJ*, *hemH*, and *FTT0673*/*prsA* intergenic mutants did not display any detectable difference in O-antigen laddering in comparison to wild type. Restoration of capsule production and LPS O-antigen laddering were observed for both the *wzy* and *manB* complemented mutants (Figure 3).

**Figure 4:**
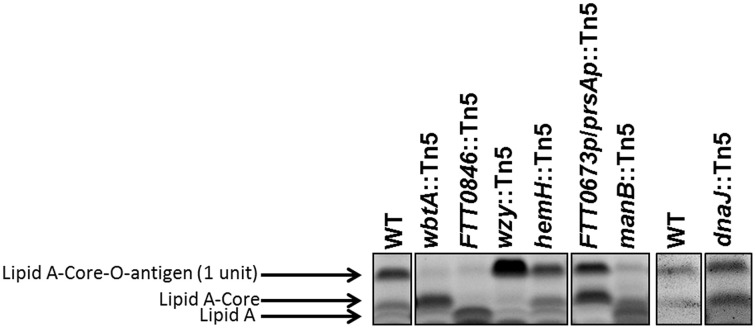
**Lipopolysaccharide core structures of mutant strains**. An SDS-PAGE gel was loaded with ~14 μg of dried material per mutant per well. The gel was stained with Emerald green, and is labeled to indicate lipid A—core—O-antigen (one unit), lipid A—core (no O-antigen subunit), or free lipid A (indicated by black arrows).

We have shown previously that mutations in the *Francisella waaY* and *waaZ* genes result in LPS core truncations, in addition to capsule and O-antigen defects (Rasmussen et al., 2014). Based upon those observations, whole cell lysates from the *dnaJ*::Tn5, *hemH*::Tn5, *manB*::Tn5, *wbtA*::Tn*5*, *FTT0846*::Tn*5*, and *FTT0673p*/*prsAp*::Tn*5* mutant strains were stained for the presence of carbohydrates to determine if the strains carried defects in the core of the LPS. Mutants in *hemH*, *dnaJ1*, *wbtA*, and *wzy* did not appear to have core defects, as the core structures appeared to be the same as the *F. tularensis* Schu S4 parent, whereas the *FTT0846* and *manB* mutants LPS core structures were either truncated or lacking (Figure 4). If the core structure is lacking for these mutants it is likely that the band observed is free lipid A.

### Determining *in vitro* growth phenotypes of mutants in human MDMs and murine BMDMs

**Figure 5:**
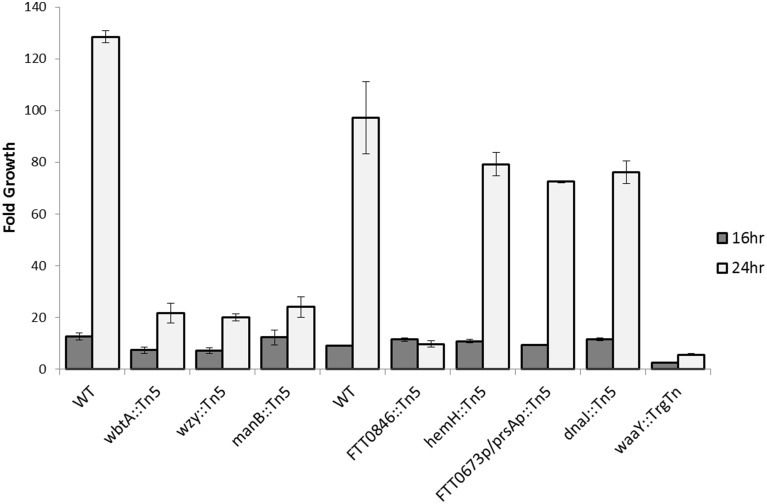
**Intracellular growth of *wbtA*, *wzy*, *manB*, *0846*, *hemH*, *dnaJ*, *waaY*, and *0673*/*prsA* intergenic mutants in MDMs**. Triplicate wells containing human macrophages were infected for 1 h with unopsonized Schu S4 or mutant bacteria, washed to remove extracellular organisms, and then lysed with 1% saponin at 1, 16, and 24 h post infection to enumerate viable bacteria. Data presented show fold growth change from 1–16 h post-infection to 1–24 h post-infection. Data are from one experiment representative of three different experiments.

Since our previous work has shown that *F. tularensis* strains defective in LPS and/or capsule O-antigen biosynthesis have growth defects in human MDMs (Lindemann et al., 2011), we examined whether the strains that are the subject of this work display a similar phenotype in either MDMs or BMDMs. Due to the serum sensitivity of the mutants, a low percentage of heat-inactivated pooled human serum was used in the tissue culture media of the MDMs to prevent serum-induced killing of the bacteria. MDMs were infected at an MOI of 100:1 with wild type Schu S4, *wbtA*::Tn*5*, *wzy*::Tn*5*, *manB*::Tn*5*, *FTT0846*::Tn*5*, *hemH*::Tn*5*, *FTT0673p*/*prsAp*::Tn*5*, *dnaJ*::Tn*5*, and *waaY*::TrgTn as a negative control. Due to the number of strains being tested, we separated the strains into two separate groups (Group 1—wild type, *wbtA*::Tn*5*, *wzy*::Tn*5* and *manB*::Tn*5* and Group 2—wild type, *FTT0846*::Tn*5*, *hemH*::Tn*5*, *FTT0673p*/*prsAp*::Tn*5* and *dnaJ*::Tn*5*) to perform human MDM infection and growth assays. Time points were taken at 1, 16, and 24 h post-infection to determine how many bacteria were present in the MDM host cells at each time point so that growth within the cells could be calculated. In contrast to the phenotype of *waaY*, *waaZ*, and *waaL* mutants, which exhibit increased uptake into MDMs (although overall growth of these strains was comparable to Schu S4 until 16 h) (Lindemann et al., 2011), the seven new mutants tested did not differ significantly in uptake or in growth rate up to 16 h post-infection (Figure 5). However, at the 24 h time point it was clear that strains with mutations in *wbtA*, *wzy*, *manB*, and *FTT0846* did not increase in number compared to the 16 h time point while wild type strain continued to replicate rapidly. This is a similar phenotype to that observed with the *waaL, waaY*, and *waaZ* mutants, as growth was limited from 16 to 48 h post-infection (Lindemann et al., 2011). Not surprisingly, the *hemH*, *dnaJ*, and the intergenic *FTT0673*/*prsA* mutants did not differ considerably from wild type for growth in MDMs, since the LPS, capsule and core sugar properties of these strains were not significantly different from the parent Schu S4 (Figure 5 and Figure S2). Since the *hemH*, *dnaJ*, and the intergenic *FTT0673*/*prsA* mutants did not have significant differences in LPS, capsule or core sugar properties, or growth in MDMs, we focused our efforts on characterizing the other mutants in more detail.

**Figure 6:**
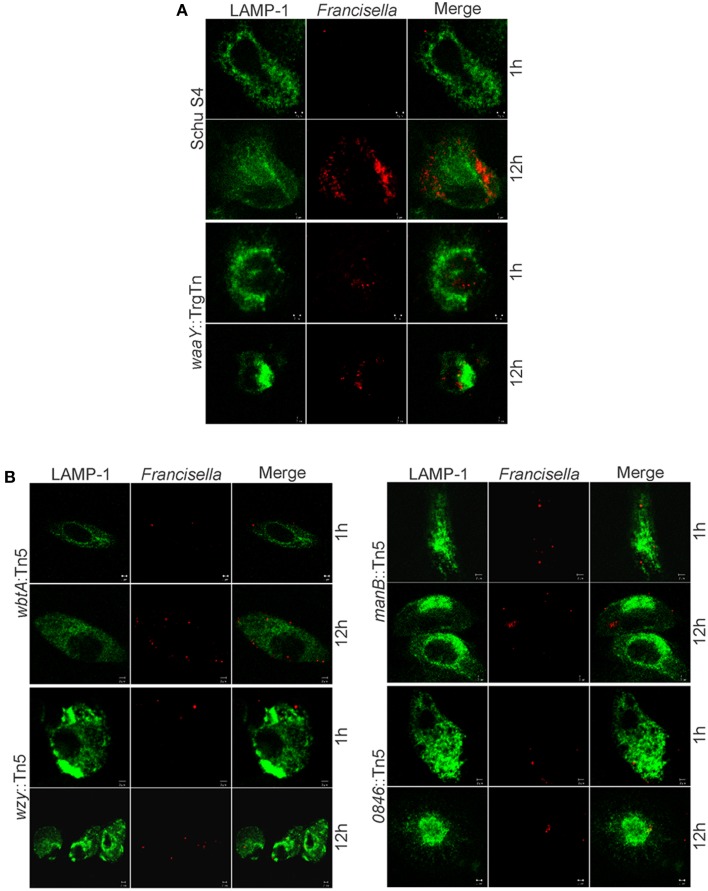
**Microscopy of infected MDMs with *wbtA*, *wzy, manB*, and *FTT0846* mutants**. Confocal images show Lamp-1 in green and bacteria in red. MDMs were infected with **(A)** wild type Schu S4 as the positive control, *waaY*::TrgTn as a negative control, and with **(B)** *wbtA*::Tn5, *wzy*::Tn5, *manB*::Tn5, or *FTT0846*::Tn5 strains at an MOI of 100:1. Samples were fixed and processed at 1 and 12 h post infection.

In addition to the intracellular MDM growth assay, mutant strains were also analyzed by confocal microscopy. Macrophages were infected at an MOI of 100:1 with wild type Schu S4 and mutant strains. The confocal microscopy data suggest that *wbtA*::Tn5, *wzy*::Tn5, *manB*::Tn5 and *FTT0846*::Tn5 strains were phagocytosed to the same extent as wild type, and did not colocalize with the Lamp-1 lysosomal marker, despite diminished intracellular growth at later stages of infection (Figure 6).

**Figure 7:**
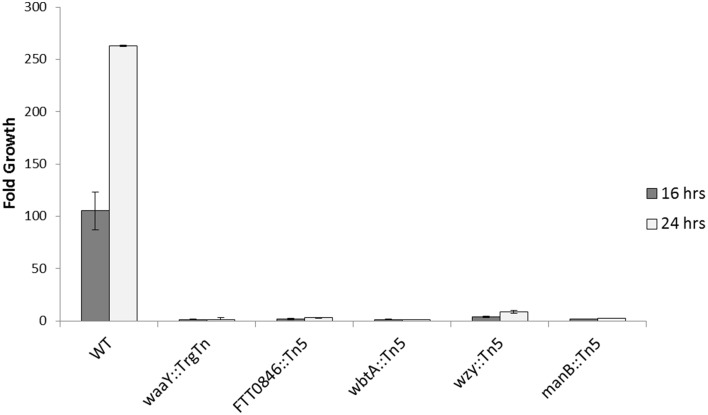
**Intracellular growth of *FTT0846*, *wbtA*, *wzy*, and *manB* mutants in BMDMs**. Murine macrophages were infected for 1 h with unopsonized Schu S4 or mutant bacteria, washed to remove extracellular organisms, and then lysed with 0.5% saponin at 1, 16, and 24 h post infection to enumerate viable bacteria. Data presented shows fold growth change from 1–16 h post infection to 1–24 h post infection. Data are from one experiment that is representative of three experiments.

Similar to the human MDM experiments, the BMDMs were infected at an MOI of 100:1, using heat-inactivated serum, and growth data was collected 1, 16, and 24 h post-infection. The data revealed that the *FTT0846*::Tn5, *manB*::Tn5, and the negative control, *waaY*::TrgTn appeared to infect BMDMs slightly better than wild type; whereas, *wbtA*::Tn5 and *wzy*::Tn5 appeared to have similar entry levels into murine BMDMs. Regardless of uptake efficiency, none of the *F. tularensis* mutants was able to replicate within the BMDMs to any significant extent over the course of the 24 h infection (Figure 7 and Figure S3). These data suggest that the internalized organisms were viable but were unable to grow in murine macrophages.

### Determination of virulence and organ dissemination in murine infections

In an effort to extend these observations and test the effects of the mutations on mouse virulence, mice were infected intranasally with *F. tularensis* Schu S4 (23 CFU), or with *F. tularensis* mutant strains, *dnaJ*::Tn*5*, *hemH*::Tn*5*, *manB*::Tn*5*, *wbtA*::Tn*5*, *wzy*::Tn*5*, *FTT0673p*/*prsAp*::Tn*5*, and *FTT0846*::Tn*5* with doses ranging from 10^1^ to 10^5^ CFU. As was observed before during murine infections with the *waaY* and *waaL* mutants (Rasmussen et al., 2014), mice infected with *manB*, *wbtA*, *wzy*, or *FTT0846* mutants displayed piloerection (ruffled fur) and lethargy as early as 24 h post-infection regardless of dose. These observations are in contrast to what was observed with mice infected with wild type, *dnaJ*::Tn5, *hemH*::Tn5, or *FTT0673p*/*prsAp*::Tn*5*. Those mice did not appear sick until 72–96 h post-infection. Mice infected with the intergenic *FTT0673/prsA*, *dnaJ*, or *hemH* mutants did not survive any of the doses tested (Table 1), indicating that these strains retain virulence similar to parent *F. tularensis* Schu S4, as mutant-infected mice died on a similar time frame as wild type. Using the Muench and Reed calculation method (Reed and Muench, 1938), we determined the i.n. LD_50_ values to be 5 × 10^3^ for *manB*::Tn5, 2.6 × 10^2^ for *wbtA*::Tn5, 1.2 × 10^4^ for *wzy*::Tn5, and 1.5 × 10^3^ CFU for *FTT0846*::Tn5 (Table 1 and Figure S4).

**Table 1:** **Murine virulence studies and experimentally determined intranasal LD_50_ values for *F. tularensis* Schu S4 and capsule and/or LPS mutants**.

**Strain**	**Infection dose (CFU)**	**Survival ratio**	**Mean TTD**	**Calc LD_50_ (CFU)**
**INTRANASAL**				
WT	23	0/5	5	<23
*dnaJ::Tn5*	1.0 × 10^2^	0/5	4.8	<100
*dnaJ::Tn5*	1.0 × 1.0^3^	0/5	4.8	
*dnaJ::Tn5*	1.0 × 10^4^	0/5	4.4	
*dnaJ::Tn5*	1.0 × 10^5^	0/5	4	
*hemH::Tn5*	1.1 × 10^2^	0/5	6	<110
*hemH::Tn5*	1.1 × 10^3^	0/5	5	
*hemH::Tn5*	1.1 × 10^4^	*0/5*	4.2	
*hemH::Tn5*	1.1 × 10^5^	0/5	4	
*man*B::Tn5	1.0 × 10^2^	5/5	>27	5 × 10^3^
*man*B::Tn5	1.0 × 10^3^	5/5	>27	
*man*B::Tn5	1.0 × 10^4^	0/5	7.6	
*man*B::Tn5	1.0 × 10^5^	0/5	6.6	
*wbtA::Tn5*	1.8 × 10^1^	4/4	>27	2.6 × 10^2^
*wbtA::Tn5*	1.8 × 10^2^	3/5	>27	
*wbtA::Tn5*	1.8 × 10^3^	0/5	17.6	
*wbtA::Tn5*	1.8 × 10^4^	0/5	7.6	
wzy::Tn5	1.2 × 10^2^	5/5	>27	1.2 × 10^4^
wzy::Tn5	1.2 × 10^3^	4/5	8, >27	
wzy::Tn5	1.2 × 10^4^	3/5	8, >27	
wzy::Tn5	1.2 × 10^5^	0/5	5.8	
*FTT0673plprsAp::Tn5*	1.4 × 10^2^	0/5	5.8	<140
*FTT0673plprsAp::Tn5*	1.4 × 10^3^	0/5	5	
*FTT0673p/prsAp::*Tn5	1.4 × 10^4^	0/5	4.8	
*FTT0673plprsAp::*Tn5	1.4 × 10^5^	0/5	4.2	
*FTTOB46::Tn5*	6.5 × 10^1^	5/5	>27	1.5 × 10^3^
*FTTOB46:*:Tn5	6.5 × 10^2^	4/5	25, >27	
*FTTOB46:*:Tn5	6.5 × 10^3^	0/5	17	
*FTTOB46::Tn5*	6.5 × 10^4^	0/5	12	

## Discussion

In this work, we identify *F. tularensis* strains that are defective in capsule and/or LPS biosynthesis by using ELISA to screen a *F. tularensis* Schu S4 transposon mutant library (7500 individual mutants) (Rasmussen et al., 2014). We identified insertions in six different genes [*waaY* (2 unique insertions), *waaL* (3 unique insertions), *wzy*, *wbtA*, *hemH*, and *FTT0846*] that we subsequently demonstrated were important in capsule biosynthesis (Rasmussen et al., 2014). The *waaY* and *waaL* genes were also identified previously in our lab from a TraSH screen in human MDMs (Lindemann et al., 2011). Re-identifying mutants in the *waaL* and *waaY* genes provided a strong level of confidence that the other mutant strains identified by ELISA were altered in capsule and/or LPS biogenesis. Since our group has previously identified and characterized the roles of *waaY* and *waaL* in capsule and LPS biosynthesis, we have used these strains as controls for the characterization of the new mutants. Consistent with involvement in capsule and LPS biosynthesis, we found that mutation of each of these genes rendered the strains more sensitive to human pooled serum (from 5-fold to 2.4 × 10^4^-fold) than the parent *F. tularensis* Schu S4. These results are similar to what is observed in other organisms when capsule and/or LPS genes are mutated (Cryz et al., 1984; Rietschel, 1984; Stawski et al., 1990; Dasgupta et al., 1994). Examination of the *hemH*::Tn*5*, *dnaJ*::Tn*5*, and *FTT0673p*/*prsAp*::Tn*5* strains revealed that they did not have defects in capsule or LPS that could detected by the assays used, nor did these three mutant strains display attenuated macrophage growth defects or *in vivo* virulence defects. While these strains were observed to have varying levels of sensitivity to human serum, the amount of serum present in the pooled human serum sensitivity assay is significantly more than in the *in vitro* cell infection assay. This likely explains why no intramacrophage growth defect was observed with these three strains. Although it is not clear why *hemH*::Tn*5*, *dnaJ*::Tn*5*, and *FTT0673p*/*prsAp*::Tn*5* were identified from the ELISA screen, it may have been that the growth conditions employed for the initial ELISA screen maximized differences in expression of capsule and/or LPS production between these strains and the wild type positive control. Besides identifying these three mutant strains of low interest, the ELISA screens identified six mutant strains of high interest that are altered in capsule and/or LPS production, of which four (*wzy*::Tn*5*, *wbtA*:Tn*5*, *manB*::Tn*5*, and *FTT0846*::Tn5) are characterized further here. Complementation of the defects in these strains was attempted several times with different vector constructs, including with a vector driving expression of the genes with the P*_gro_* promoter. Thus, we conclude that for the *FTT0846*::Tn5 and *wbtA*::Tn5 mutant strains that the phenotypes observed are either due to disruption of the gene in which the transposon is inserted or to downstream polar effects.

In examining the annotated functions of *wzy*, *wbtA*, *manB* and *FTT0846*, it is unclear how the annotated function of the *FTT0846* gene product is relevant to LPS and/or capsule biosynthesis. *FTT0846* is predicted to encode a deoxyribodipyrimidine photolyase, a class of proteins that are involved in sensing blue light to repair DNA damage. However, it has been postulated that bacterial photolyases can be involved in virulence by regulating the transcription of genes either directly or indirectly (Gaidenko et al., 2006; Purcell et al., 2007; Swartz et al., 2007; Purcell and Crosson, 2008; Cahoon et al., 2011). It is possible that protein produced by *FTT0846* is involved in some way in the regulation of LPS core assembly or synthesis, due to the degree of serum sensitivity, a MDM growth defect, and the truncated core observed in the *FTT0846* mutant. Future work will be aimed at exploring these possibilities in detail.

For the three other mutants identified from the screens, *wbtA*::Tn*5*, *wzy*::Tn*5*, and *manB*::Tn*5*, there are clear connections to capsule and LPS synthesis. WbtA is a dTDP-glucose 4,6-dehydratase and has been shown in LVS (Raynaud et al., 2007; Sebastian et al., 2007) and other pathogenic organisms such as *Pseudomonas aeruginosa*, *Bordetella pertussis*, and *Yersinia enterocolitica* to be essential for complete LPS biosynthesis (Creuzenet and Lam, 2001). Specifically, WbtA orthologs are involved in the formation of 6-deoxy sugars (Creuzenet et al., 2000; Creuzenet and Lam, 2001), and the first sugar in the tetrasaccharide repeat of *F. tularensis* is a 6-deoxy sugar, QuiNAc (Vinogradov et al., 2002). We have presented *wbtA* mutant data that is consistent with the presumed function of this gene in *F. tularensis*, as the serum sensitivity of the mutant is increased and a complete LPS core is present, but capsule and LPS-associated O-antigens are not produced as we could not detect these structures in our assays. Interestingly, the glycosylated proteins that we have reported to be present in a *waaY* mutant strain (Jones et al., 2014), are not present in the *wbtA* mutant, providing further support for the idea that the *wbtA* gene product is important for producing complete O-antigen subunits (data not shown).

The Wzy protein is annotated as an O-antigen polymerase and this function was confirmed by Kim et al. (2010) in LVS. The LVS *wzy* mutant has normal LPS core, but has only one O-antigen tetrasaccharide repeat attached to core (Kim et al., 2010). From our data, the Schu S4 *wzy* mutant was observed to have a similar phenotype (intact core with only one O-antigen tetrasaccharide repeat). In addition, we observed that this strain lacked O-antigen reactive capsule and displayed an increase in sensitivity to serum.

ManB is a phosphomannose mutase that has been shown in *F. novicida* to be important for LPS core production (Lai et al., 2010). The Schu S4 *manB*::Tn*5* has a phenotype similar to the *F. novicida manB* mutant. Experiments to visualize LPS laddering in the Schu S4 *manB* mutant revealed that it was absent. Surprisingly, we observed that both the *FTT0846* and *manB* mutants produced higher than normal molecular weight capsules albeit in reduced amounts. An unanticipated result from this work has been that mutants with defects in the *F. tularensis* core sugar assembly pathway do not produce normal LPS O-antigen laddering, yet they still produce capsule which appears larger in size than its wild-type counterpart. An exception to this observation is the *F. tularensis waaY*::TrgTn mutant strain that does not produce LPS O-antigen laddering or capsule. However, it should be noted that this strain has a polar insertion which also disrupts the downstream gene, *waaZ*. A goal of the work by our group is to understand this phenomenon in more detail.

When the *manB*, *wzy*, *wbtA* and *FTT0846* mutants were used to infect MDMs, no observable differences in intracellular growth were detected at 1 h post–infection or at 16 h post–infection compared to the parent *F. tularensis* Schu S4. However, by 24 h post–infection there were substantial differences in growth between the mutant strains and the wild type strain, indicating that replication of the mutants in MDMs at later stages of infection is impaired. These phenotypes are similar to those we reported previously for the *F. tularensis* Schu S4 *waaY*, *waaZ*, and *waaY* O-antigen and capsule mutants (Lindemann et al., 2011). The mutants are even less able to grow within murine macrophages as the *manB*, *wzy*, *wbtA*, and *FTT0846* mutants displayed almost no growth in bone marrow derived macrophages during the 24 h infection experiment.

In addition to *in vitro* experiments, virulence studies were performed by intranasally infecting mice with the *manB*, *wzy*, *wbtA* or *FTT0846* mutants, and their respective LD_50_ values were calculated to be 5.0 × 10^3^-fold, 1.2 × 10^4^-fold, 260-fold, and 1.5 × 10^3^-fold more than the previously published wild type theoretical LD_50_ of ~1 CFU (Kim et al., 2012). Mice that were infected with *manB*::Tn5, *wbtA*::Tn5, *wzy*::Tn5 or *FTT0846*::Tn5 displayed ruffled fur (piloerection) within 24–48 h post-infection, suggestive of a gross inflammatory response, similar to what we observed for mice infected with the *waaY*::TrgTn or *waaL*::TrgTn strains (Rasmussen et al., 2014). These data are consistent with the *in vitro* cell growth results in human MDMs and murine BMDMs, in that these mutations impair intracellular growth when compared to Schu S4 over 24 h. In contrast, the *hemH*::Tn*5*, *dnaJ*::Tn*5*, and *FTT0673p*/*prsAp*::Tn*5* strains did not have a growth phenotype in macrophages, did not elicit gross inflammatory responses in mice, and did not show an increase in their LD_50_ values for mice compared to wild type.

As mentioned previously, work has been done with *F. tularensis* LVS *wzy* and *wbtA* mutants. Although our data are in agreement with the O-antigen defects, serum sensitivity, and intramacrophage growth defect reported by other groups with LVS mutants (Raynaud et al., 2007; Sebastian et al., 2007; Thomas et al., 2007; Kim et al., 2010), we observed significantly less diminution of virulence in our i.n. infection experiments using the Schu S4 mutant strains. Sebastian et al. (2007) and Kim et al. (2012) showed that mice infected with either a LVS *wbtA* or *wzy* mutant survived a 10^7^ CFU i.n. infection, which is a 20,000-fold increase in the LVS LD_50_. However, we observed 26-fold and 1200-fold increase in the LD_50_ values, respectively, for the Schu S4 *wbtA* and Schu S4 *wzy* mutants intranasally infected into mice. These data highlight the importance of considering strain background in understanding the role and contribution to virulence of different *Francisella* factors.

**Figure 8:**
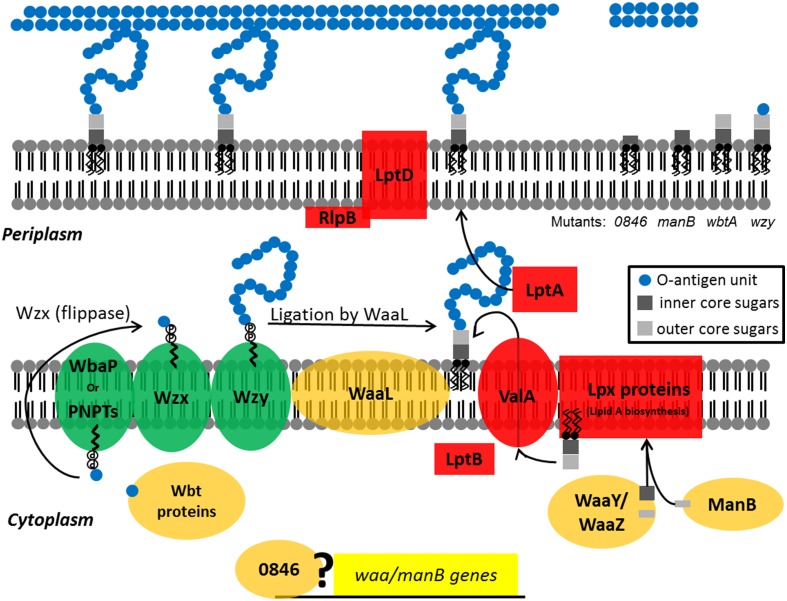
**Diagram depicting the activities of the F. tularensis WaaY, WaaZ, WaaL, ManB, 0846, Wzy, and WbtA proteins in LPS biosynthesis**. LPS is synthesized as two separate components, which are (1) lipid A-core sugars and (2) polymers of O-antigen subunits. O-antigen sugars are synthesized in part by WbtA in the cytoplasm and assembled onto a lipid carrier, the undecaprenol pyrophosphate that is associated with WbaP or PNPT, polyisoprenyl-phosphate hexose-1-phosphate transferase and N-acetylhexosamine-1-phosphate transferase, respectively. The Wzx protein flips the charged lipid carrier, with the assembled O-antigen unit, through the inner membrane from the cytoplasm to the periplasmic space. Repeating O-antigen subunits are polymerized on the lipid carrier by the Wzy protein. The lipid A portion of LPS is synthesized by a number of Lpt and Lpx proteins at the inner leaflet of the inner bacterial membrane. WaaY, WaaZ, and ManB proteins modify or transfer core sugars to the lipid A molecule as they help assemble the core structure of LPS. ValA and LptB transports the lipid A-core sugars to the outer leaflet of the inner membrane where O-antigen subunits are added by the O-antigen ligase, WaaL, and the mature molecule is chaperoned to the outer membrane by LptA and LptD/RlpB (Kim et al., 2010). Possibly the FTT0846 protein is involved in regulation of core sugar genes. *F. tularensis* mutants that are disrupted in FTT0846 or manB production are depicted with truncated cores whereas the lipid-core sugar structure of the wbtA mutant has a complete core, but no O-antigen. The wzy mutant has a complete core and a single O-antigen tetrasaccharide unit.

**Figure 9:**
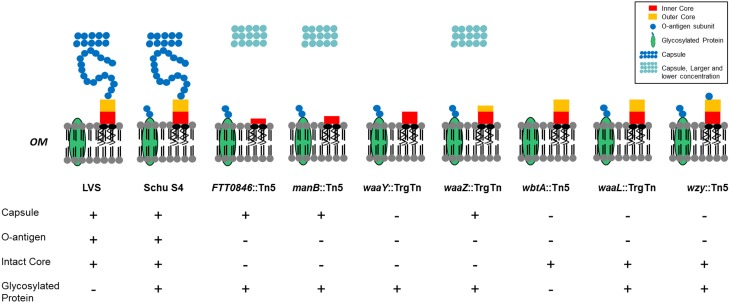
**Phenotypes of capsule and/or O-antigen mutants**. Properties of the *F. tularensis* mutants include the presence or absence of capsule, O-antigen, intact core, or O-antigen associated glycosylated proteins (Jones et al., 2014). FTT0846::Tn5, manB::Tn5, and waaZ::TrgTn are shown with a capsule structure that is higher in molecular weight but reduced in concentration when compared to wild type.

In summary, we present further evidence that the LPS and capsule structures of *F. tularensis* are essential components of its virulence strategy. Mutations in *wzy*, *wbtA*, *manB*, and *FTT0846* affect capsule production, or LPS core and O-antigen structures (Figure 8). In general, mutants with defects in LPS or capsule are sensitive to serum, do not show robust growth after 16 h within human macrophages nor do they grow in murine macrophages, and are attenuated in a murine infection model. In total, our research group has identified seven mutant strains that display varying differences in LPS core structure, LPS-associated O-antigen, capsule, and O-antigen glycosylated proteins in comparison to Schu S4 (Figure 9). These strains are of interest as they grow poorly in macrophages or mouse in an infection model which demonstrates that they are attenuated for virulence. We believe that our data demonstrate that these mutant strains are being detected by the host, which may be a critical first step toward development of a vaccine to an immunologically invisible pathogen. Future studies will focus on what pathway(s) are activated and the degree to which each mutant stimulates a response. The loss of capsule/O-antigen structures allows some level of host recognition of the pathogen, but clearly does not render the strains avirulent. As such, these strains may be reasonable models for genetic dissection of other components of *Francisella* virulence. In addition, we believe that these strains may be an appropriate place to begin development of a viable, defined vaccine for tularemia.

### Conflict of interest statement

The authors declare that the research was conducted in the absence of any commercial or financial relationships that could be construed as a potential conflict of interest.

## Supplementary material

Figure S1 **Growth curves of *F. tularensis* Schu S4 and each of the mutants**. Every 2 h samples were taken from each of the cultures and the OD_600_ was determined.

Figure S2 **Intracellular growth of *wbtA*, *wzy*, *manB*, *0846*, *hemH*, *dnaJ*, *waaY*, and *0673*/*prsA* intergenic mutants in MDMs**. Triplicate wells containing human macrophages were infected for 1 h with unopsonized Schu S4 or mutant bacteria, washed to remove extracellular organisms, and then lysed with 1% saponin at 1, 16, and 24 h post infection to enumerate viable bacteria. Data presented show the average number of bacteria recovered for each strain at 1, 16, and 24 h post-infection. Data are from one experiment representative of three different experiments. **(A)** Data for the wild type, *manB*::Tn*5*, *wzy*::Tn*5*, *wbtA*::Tn*5 F. tularensis* strains. **(B)** Data for the wild type, *FTT0846*::Tn*5*, *hemH*::Tn*5*, *FTT0673p*/*prsAp*::Tn*5*, *dnaJ*::Tn*5*, *waaY*::Tn*5 F. tularensis* strains.

Figure S3 **Intracellular growth of *FTT0846*, *wbtA*, *wzy*, and *manB* mutants in BMDMs**. Murine macrophages were infected for 1 h with unopsonized Schu S4 or mutant bacteria, washed to remove extracellular organisms, and then lysed with 0.5% saponin at 1, 16, and 24 h post-infection to enumerate viable bacteria. Data presented shows the average number of bacteria recovered for each strain at 1, 16, and 24 h post-infection. Data are from one experiment that is representative of three experiments.

Figure S4 **Survival curves of BALB/c mice infected with *F. tularensis* Schu S4 wild type or mutant strains carrying transposon insertions in *dnaJ*, *hemH*, *manB*, *wzy*, *wbtA*, *FTT0846* and between *FTT0673* and the *prsA* gene**. Mice were infected with 23 cfu of *F. tularensis* Schu S4 as a positive control for virulence. Four groups of mice were infected for each of the indicated *F. tularensis* mutant strain at four different doses that increased 10-fold. Mice were examined daily and the number of mice that were alive each day from each group was recorded and is shown on the graph. Mice infected with strains with mutations that did not significantly alter virulence were typically dead at day 5 or day 6. Mice infected with attenuated strains were followed up to 40 days post-infection depending upon signs of illness such as piloerection, hunching and lethargy. Some infected groups of mice became ill at significantly delayed time points and those groups of mice were followed for extended periods of time. Ultimately all mice were sacrificed according to protocol and disposed of in accordance with BSL-3 regulations.
