# Supplementary figures and images for: Characterization of Francisella tularensis Schu S4 mutants identified from a transposon library screened for O-antigen and capsule deficiencies
Source: Front Microbiol. 2015 May 5;6:338. doi: 10.3389/fmicb.2015.00338 (PMC4419852; doi:10.3389/fmicb.2015.00338)

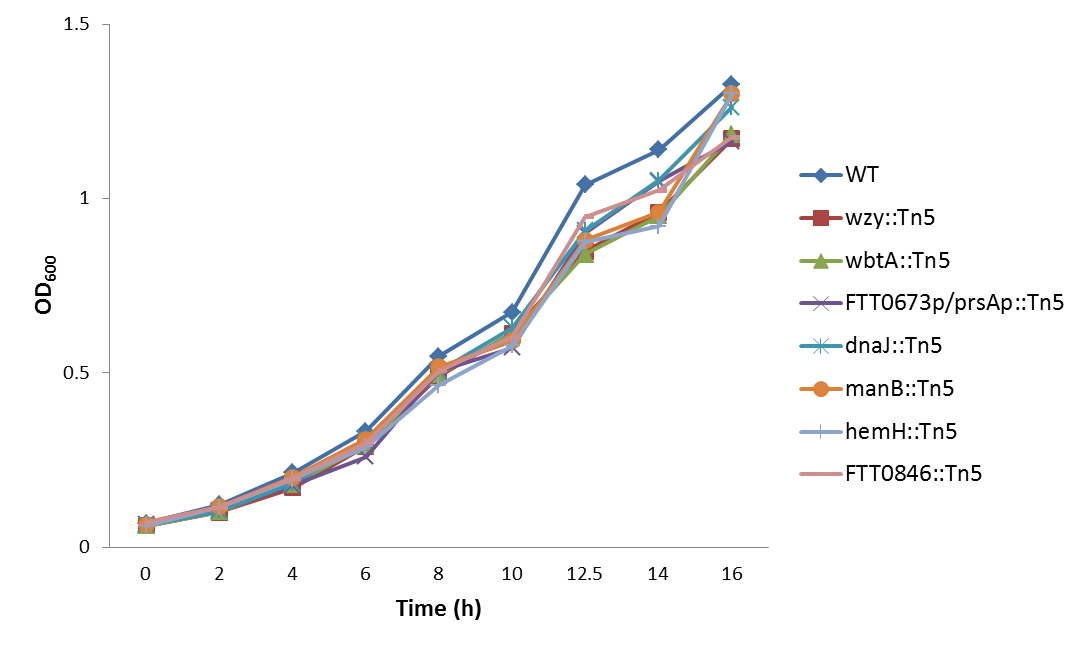

Supplement: Figure S1 — Growth curves of F. tularensis Schu S4 and each of the mutants. Every 2 h samples were taken from each of the cultures and the OD600 was determined. [file Presentation_1.ZIP › Supplementary Material/131951_Jones_Image_1.TIF]

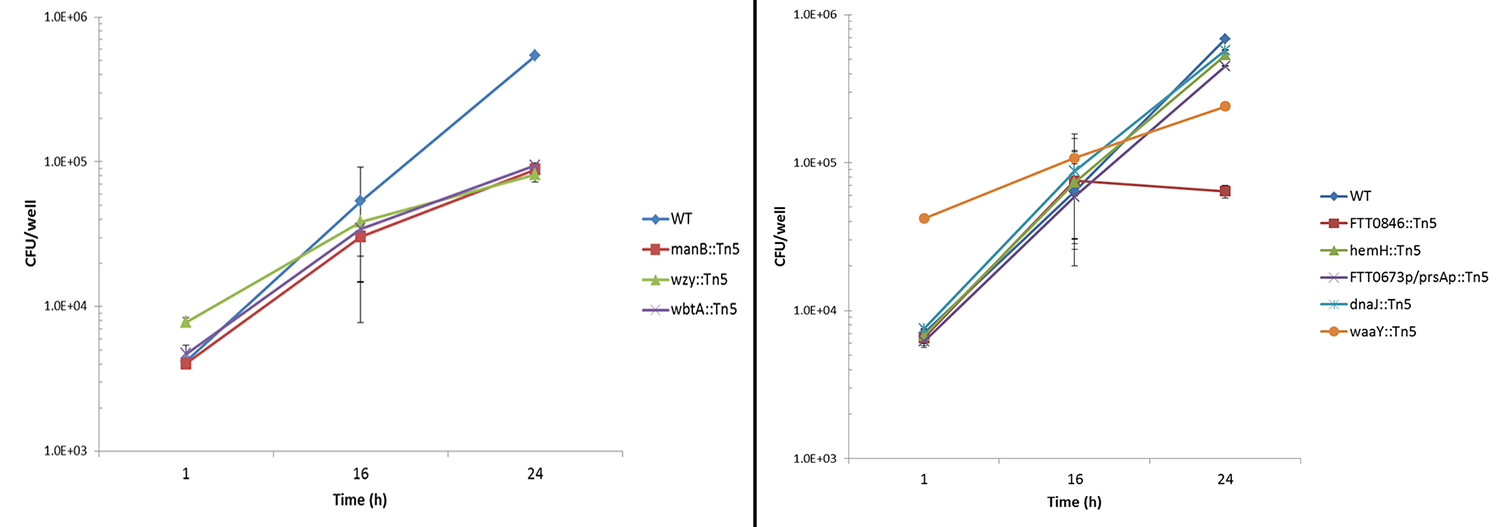

Supplement: Figure S1 — Growth curves of F. tularensis Schu S4 and each of the mutants. Every 2 h samples were taken from each of the cultures and the OD600 was determined. [file Presentation_1.ZIP › Supplementary Material/131951_Jones_Image_2.TIF]

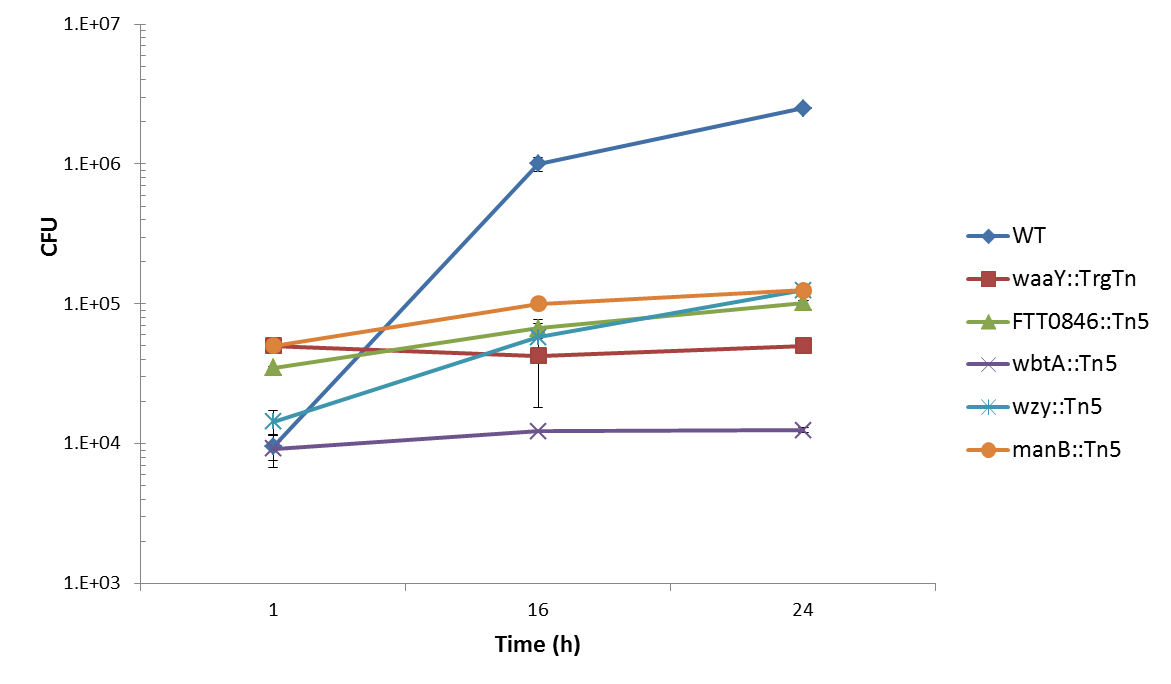

Supplement: Figure S1 — Growth curves of F. tularensis Schu S4 and each of the mutants. Every 2 h samples were taken from each of the cultures and the OD600 was determined. [file Presentation_1.ZIP › Supplementary Material/131951_Jones_Image_3.TIF]

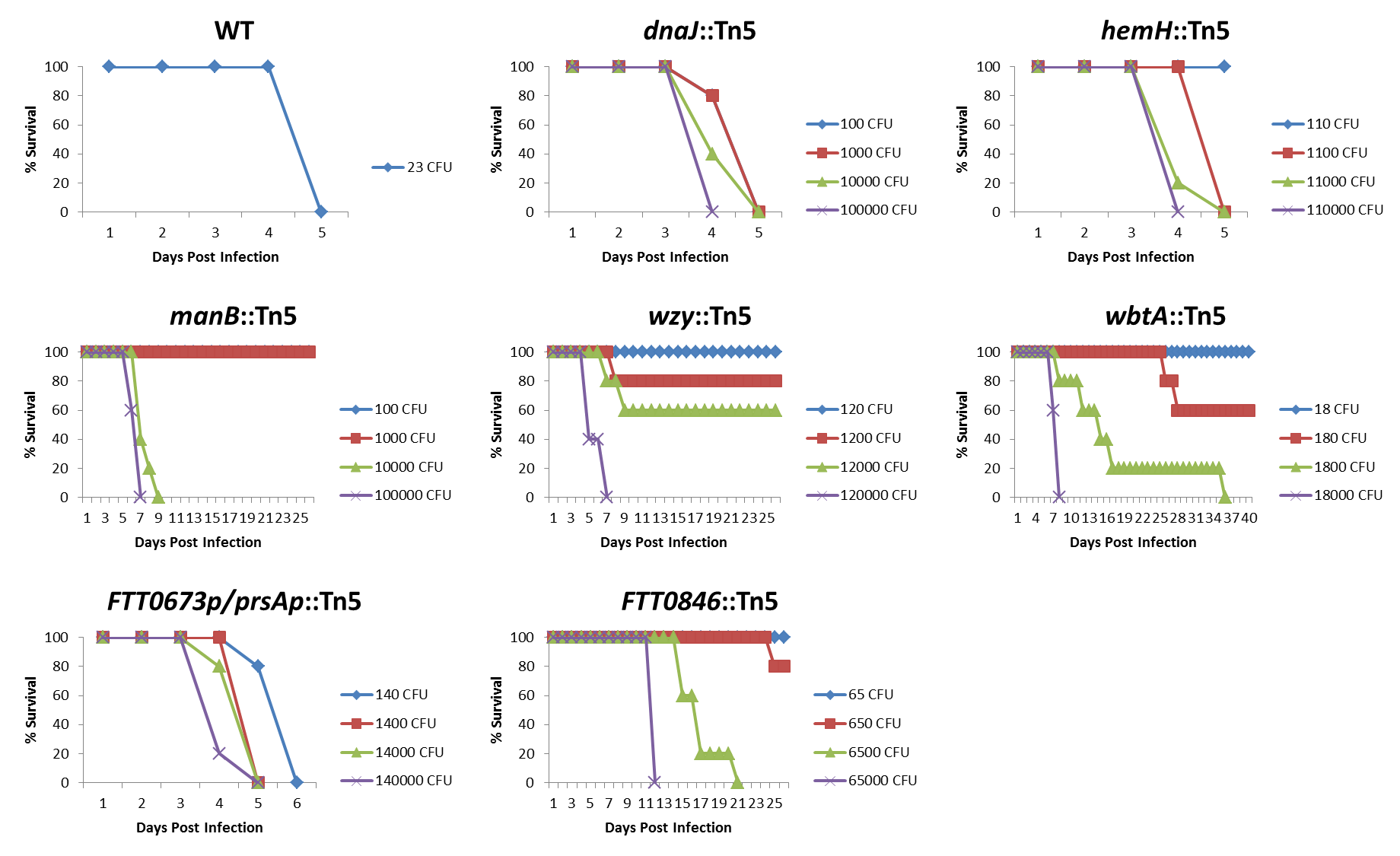

Supplement: Figure S1 — Growth curves of F. tularensis Schu S4 and each of the mutants. Every 2 h samples were taken from each of the cultures and the OD600 was determined. [file Presentation_1.ZIP › Supplementary Material/131951_Jones_Image_4.TIF]
